# Prevalence of *bla*_OXA-48_ and other carbapenemase encoding genes among carbapenem-resistant *Pseudomonas aeruginosa* clinical isolates in Egypt

**DOI:** 10.1186/s12879-024-10123-7

**Published:** 2024-11-11

**Authors:** Wedad M. Abdelraheem, Doaa Elzaeem Ismail, Safaa S. Hammad

**Affiliations:** 1 Medical Microbiology and Immunology department, Faculty of Medicine, Minia University, Minia, Egypt; 2 Department of Clinical Pathology, Faculty of Medicine, Minia University, Minia, Egypt

**Keywords:** *bla*_OXA−48_ gene, carbapenem resistance, class 1 integron, MDR, *P. aeruginosa*, Tn*1999*

## Abstract

**Background:**

Resistance to carbapenem, the last line of treatment for gram-negative bacterial infections has been increasing globally and becoming a public health threat. Since integrons may aid in the transmission of resistance genes, the purpose of this study was to detect the frequency of class 1, 2, and 3 integrons as well as carbapenem-resistant genes in clinical isolates of *P. aeruginosa* that are resistant to carbapenem.

**Methods:**

This study was carried out on 97 clinical isolates of *P. aeruginosa* isolated from wound and urine samples. The antimicrobial susceptibility for all isolates was tested by the disc diffusion method. The presence of integrons and carbapenem-resistant genes among carbapenem-resistant *P. aeruginosa* isolates was evaluated by conventional PCR.

**Results:**

The antimicrobial resistance rate among *P. aeruginosa* clinical isolates was high, with imipenem resistance in 58.8% of the studied isolates. In this study, 86% of the carbapenem-resistant *P. aeruginosa* isolates carry carbapenemase genes, with *bla*_VIM_ being the most common gene followed by the *bla*_OXA−48_ gene. Class 1 and class 2 integrons were reported in 37 (64.9%) and 10 (17.5%) of the tested carbapenem-resistant *P. aeruginosa* isolates, respectively.

**Conclusion:**

Our data reported a high prevalence of class 1 integrons in carbapenem-resistant *P. aeruginosa* clinical isolates, suggesting the important role of integrons in carbapenem-resistant gene transfer among such isolates.

**Supplementary Information:**

The online version contains supplementary material available at 10.1186/s12879-024-10123-7.

## Introduction

Carbapenems are the last therapeutic option for treating multidrug-resistant (MDR) bacterial infections. However, the continuous increase in carbapenem resistance becomes a global public health threat as they mediate resistance to all β-lactam antibiotics and most other antibiotic classes except colistin, some aminoglycosides, and tigecycline [1]. Carbapenem resistance is most commonly due to the acquisition of resistance genes encoding carbapenemases (carbapenem hydrolyzing β-lactamases). Carbapenem-hydrolyzing β-lactamases belonging to molecular class A (e.g., KPC, GES, IMI, SME), class B (e.g. IMP, VIM, NDM, GIM), and class D (e.g. OXA-23 and OXA-48) [2].

The *bla*_OXA−48_, a class D carbapenemase, is of major concern due to its difficulty in detection and its association with treatment failure. They were first isolated in Istanbul, Turkey, in 2001in carbapenem-resistant *Klebsiella pneumoniae*. Genes encoding these carbapenemases are usually found in mobile genetic elements and are easily transferable by plasmids [3]. It has been demonstrated that *bla*_OXA−48_ was part of a composite transposon named Tn*1999* and made of two copies of IS*1999* bracketing this gene [4]. Integrons, one of the mobile genetic elements, are responsible for the spread of many antibiotic resistance genes including carbapenems resistance genes [5]. They play an important role in the dissemination and evolution of antibiotic-resistant genes due to their ability to capture and integrate antibiotic resistance genes, particularly in Gram-negative clinical isolates [6]. Based on the sequence of the integrase (intI) gene, five classes of integrons (class 1, 2, 3, 4, and 5) have been identified in Gram-negative bacteria, but only three classes (class 1, 2 and 3) are known to be responsible for multi-drug resistance (MDR) [7]. Class 1 integrons are the most important class among clinical isolates and are considered a major contributor to the high prevalence of MDR in these isolates [8]. *Pseudomonas aeruginosa* (*P. aeruginosa*) is an opportunistic Gram-negative bacillus and a causative agent for important community- and hospital-acquired infections [9]. Carbapenem-resistant *P. aeruginosa* strains are a growing serious threat to human health in hospitals, especially in patients who use medical devices such as catheters and ventilators [10]. Therefore, this study aimed to investigate the frequency of class 1, 2, and 3 integrons& and carbapenemases encoding genes among MDR and carbapenem-resistant *P. aeruginosa* clinical isolates.

## Methods

This study was performed during the period from April 2023 to June 2024 in the Department of Medical Microbiology and Immunology, Faculty of Medicine, Minia University, Egypt. Nighty- seven non-duplicated, clinical *P. aeruginosa* isolates were recovered from different inpatients and outpatients complaining of UTI, Wound infection, or burn wound infections. In Minia University hospitals, 300 samples were collected from patients at gynecological, General, and Plastic surgery departments.

### Bacterial isolation and identification

Samples were streaked on the cetrimide and MacConkey agar (Oxoid, England), After overnight incubation at 37 °C, isolated colonies were identified by colony morphology, microscopic examination, and biochemical reactions as described previously [11]. All identified strains were further confirmed as *P. aeruginosa* by detection of the 16s rRNA gene (accession no: NR_026078) by conventional PCR using specific primers (Supplementary Table S1). All confirmed strains were stored in glycerol (20%), and then frozen at -20 °C for further tests.

## Antibiotic susceptibility pattern of isolates

### Antimicrobial susceptibility testing

Antibiotic susceptibility testing was performed on all isolates by the disc diffusion method on Muller-Hinton agar (Oxoid, England), using the following antimicrobial discs: Cefepime (30 µg), Ceftazidime (30 µg), Gentamycin (10 µg), Amikacin (30 µg), Levofloxacin (5 µg). Imipenem (10 µg), Piperacillin-Tazobactam (100/10µg). All discs were obtained from Oxoid Company. The results were interpreted following CLSI, 2022 [12].

### Detection of carbapenemase encoding genes and integrons by conventional PCR

#### DNA extraction

The DNA of the studied *P. aeruginosa* isolates was extracted using the boiling method by the heat shock technique [13]. Briefly, centrifugation of 1.5 ml overnight bacterial culture broth at 5,000 rpm was done for 3 min in a microtubes centrifuge (Hamburg, Germany). The supernatant was discarded carefully, and the pellet was resuspended in 200 µl sterile water and mixed by pipetting. The tubes were subjected to boiling in a water bath at 100 °C for 8 min, then immediately shocked by ice-cooling for 20 min. After centrifugation at 5,000 rpm for 3 min, the supernatant with the genomic DNA was transferred into a new tube for subsequent PCR amplification. The presence of DNA was confirmed by gel electrophoresis using loading dye (Thermo Scientific-EU-Lithuania). Extracted DNA was stored at -20 ˚C until used for PCR test.

#### Conventional PCR

The following carbapenemase-encoding genes in carbapenem resistant and carbapenem sensitive isolates were tested: class A β-lactamases genes: *bla*_KPC_, *bla*_SME_, and *bla*_GES_; class B metallo-β-lactamases genes: *bla*_IMP_, *bla*_VIM_, and *bla*_NDM_; and class D oxacillinases genes: *bla*_OXA−48_ by conventional PCR using gene-specific primers [14–16]. The presence of class 1, 2, and 3 integrons in carbapenem-resistant and carbapenem-sensitive isolates was assessed using integrase-specific primers, *intI1*,* intI2*, and *intI3*, respectively [17]. The amplification reactions were carried out in a Biometra UNO II thermal cycler (Goettingen, Germany). All PCR reactions were performed in 20 µl volumes containing: 10 µl of PCR master mix COSMO PCR Hot Start RED Master Mix (Willowfort -Birmingham -UK), 1 µl of each primer (10 pmol/µl), 6 µl nuclease-free water, and 2 µl of the extracted DNA.

Amplification reactions were under the following conditions: initial denaturation at 94 ˚C for 10 min, then 30 amplification cycles (denaturation for 30s at 94˚C, annealing temperature varies according to different genes, and extension for 30s at 72˚C), and a final extension at 72˚C for 5 min. The primer sequences used in this study, annealing temperatures, and the product size are listed in Supplementary Table S1. Amplified PCR products were analyzed by electrophoresis in 1.5% agarose gel at 80 V for 45 min in TBE buffer containing ethidium bromide and visualized under UV trans-illumination.

#### Integron 1 sequencing

Ten representative isolates contained 3 carbapenemase encoding genes previously identified as being positive for *integrase 1* gene were further tested using the primers 5′-CS and 3′‐CS [18] (Supplementary Table S1), designed to anneal to the 5′ and the 3′ conserved segments and to amplify the entire integron cassette insertion region. The purified PCR amplicons of the tested isolates were sequenced by an ABI PRISM sequencer (Applied Biosystems, CA). The resulting sequences were analyzed using the BLAST program of the NCBI (accession number: AF263519, AJ969234, EF184215, and DQ984668).

### Studying the presence of transposon Tn*1999* among carbapenem resistant *P. aeruginosa* clinical isolates

Conventional PCR was performed to analyze the presence of transposon (Tn*1999*) among carbapenem-resistance *P. aeruginosa* clinical isolates by designing specific primers targeting IS*1999* (accession number: AY236073) that flanking the *bla*_OXA−48_-gene in Tn*1999* transposon (Supplementary Table S1).

### Statistical analysis

Data analyses were performed by SPSS software (version 23) and proportions were compared using the Chi-square test to determine the significant differences. Differences were considered significant at P-value < 0.05. Data are presented as frequency and percentage for categorical variables.

## Results

In this descriptive study, 97 clinical isolates of *P. aeruginosa* were isolated from 300 wound and urine samples. The number of *P. aeruginosa* isolates recovered from wound infections was 60 (61.9%) and from urinary tract infections was 37 (38.1%). Forty-five and fifty-two of the isolates were from inpatients and outpatients, respectively.

### Antimicrobial susceptibility testing

Antimicrobial susceptibility testing was done by disc diffusion method. The overall resistance rate among isolates was high. The resistance rate among *P. aeruginosa* isolates to the following antimicrobials: Ceftazidime, Cefepime, Gentamycin, Amikacin, Levofloxacin, Imipenem, and Piperacillin-Tazobactam was: 77.3%, 55.7%, 38.1%, 51.5%, 50.5%, 58.8%, 67.0%, respectively.

Table 1 shows no significant difference (P-value ˃ 0.05) in the antibiotic resistance pattern between isolates from inpatients and outpatients, except for amikacin (P-value = 0.01). Table 2 shows a significant difference (P-value ˂ 0.05) in the resistance pattern of Gentamycin, Imipenem, and Piperacillin-Tazobactam between urinary isolates and isolates recovered from wound samples.

**Table 1:** Antibiotic resistance pattern of *P. aeruginosa* isolates of inpatients and outpatients

Antibiotics	Total (*N* = 97)	Inpatient (*N* = 45)	Outpatient (*N* = 52)	*P*-value
	*N* (%)			
**Ceftazidime**	75 (77.3)	33 (56.0)	42 (68.5)	0.34
**Cefepime**	54 (55.7)	28 (44.0)	26 (42.6)	0.23
**Gentamycin**	37 (38.1)	20 (34.0)	17 (37.0)	0.26
**Amikacin**	50 (51.5)	29 (40.0)	21 (50.0)	0.01*
**Levofloxacin**	49 (50.5)	21 (28.0)	28 (40.7)	0.48
**Imipenem**	57 (58.8)	26 (44.0)	31 (50.0)	0.86
**Piperacillin –Tazobactam**	65 (67.0)	30 (44.0)	35 (48.1)	0.94

* Significant level of P-value is < 0.05

* P-value was calculated by using Chi-square test

**Table 2:** Antibiotic resistance pattern of *P. aeruginosa* urinary and wound isolates

Antibiotics	Total isolates (*N* = 97)	Wound isolates (*N* = 60)	Urine isolates (*N* = 37)	*P*-value
	*N* (%)			
**Ceftazidime**	75 (77.3)	46 (76.7)	29 (78.4)	0.84
**Cefepime**	54 (55.7)	33 (55.0)	21 (56.8)	0.86
**Gentamycin**	37 (38.1)	18 (30.0)	19 (51.4)	0.03*
**Amikacin**	50 (51.5)	27 (45.0)	23 (62.2)	0.10
**Levofloxacin**	49 (50.5)	32 (53.3)	17 (45.9)	0.47
**Imipenem**	57 (58.8)	42 (70.0)	15 (40.5)	0.00*
**Piperacillin –Tazobactam**	65 (67.0)	45 (75.0)	20 (54.1)	0.03*

* Significant level of P-value is < 0.05

* P-value was calculated by using Chi-square test

### Distribution of carbapenemases encoding genes and integron genes among carbapenem-resistant and carbapenem-sensitive isolates

Conventional PCR detected carbapenemases encoding gene and class 1, 2, and 3 integrons genes among *P. aeruginosa* isolates (*N* = 97) (Supplementary Figure S1 and S2). Out of 97 *P. aeruginosa* isolates, 57 (58.8%) were resistant to carbapenem and 40 (41.2%) were sensitive to carbapenem. Among carbapenem-resistant *P. aeruginosa* isolates, 49 (86%) were carbapenemase producers. The *bla*_VIM_, *bla*_OXA−48_, *bla*_KPC_, *bla*_IMP_, *bla*_GES,_ and *bla*_NDM_ genes were detected in 33 (57.9%), 29 (50.8%), 13 (22.8%), 8 (14%), 3 (5.2%) and 2(3.5%) of the carbapenem-resistant *P. aeruginosa* isolates, respectively. Moreover, 29 isolates (50.8%) carry more than one carbapenemase-encoding gene. None of the isolates harbored the *bla*_SME_ gene. There are 5 isolates only among the carbapenem-sensitive *P. aeruginosa* isolates (12.5%) that were positive for the tested carbapenemase encoding genes, 4 (10%) were positive for *bla*_VIM_ and 1 (2.5%) was positive for *bla*_KPC_ genes.

The PCR results showed that the prevalence of integron genes was higher among isolates that were resistant to carbapenem than isolates that were sensitive to carbapenem with a significant P value (˂0.05). Thirty-seven (64.9%) and ten (17.5%) of the carbapenem-resistant *P. aeruginosa* isolates were identified as positive for class 1 and class 2 integrons, respectively. The remaining 10 carbapenem-resistant isolates don’t contain any integron genes. While; 16 (40%) and 2 (5%) of the carbapenem-sensitive *P. aeruginosa* isolates were identified as positive for class 1 and 2 integrons, respectively. Class 3 integrons were not found among the studied isolates.

Out of 49 carbapenemases encoding genes positive carbapenem-resistant isolates, 35 (71.4%) were integron positive (32 were integron 1 positive, and 3 were integron 2 positive) and the remaining 14 (28.6%) isolates were integron negative with a significant p-value of 0.001. Table 3 shows the distribution of carbapenem-resistant *P*. *aeruginosa* isolates positive for carbapenemase encoding genes, class 1 and 2 integrons according to specimen type, patient type, and MDR pattern of the isolates. There was no significant association between the presence of carbapenemase encoding genes, class 1, and 2 integrons in carbapenem-resistant *P*. *aeruginosa* isolates with the sample or the patient type (*p* > 0.05). The prevalence of carbapenemase encoding genes and class 1 integrons was significantly higher in MDR *P*. *aeruginosa* versus non-MDR isolates (93.6% vs. 50% and 74.5% vs. 10%, respectively; p-value = 0.00) as shown in Table 3. On the other hand, the antibiotic resistance pattern to most studied antibiotics of the integron-positive carbapenem resistant isolates was significantly higher (p ˂0.05) than the integron-negative carbapenem resistant isolates as shown in Table 4.

**Table 3:** The distribution of carbapenemase-encoding genes, class 1 and 2 integrons among carbapenem-resistant *P. aeruginosa* isolates according to specimen type, patient type, and MDR pattern.

Gene	Specimen type			Patient type			MDR pattern		
	Wound (*N* = 42)	Urine (*N* = 15)	*P* ^1^	Outpatient (*N* = 31)	Inpatient (*N* = 26)	*P* ^2^	MDR (*N* = 47)	Non MDR (*N* = 10)	*P* ^3^
***bla***_**VIM**_	27 (64.3%)	6 (40%)	0.2	20 (64.5%)	13 (50%)	0.4	31 (66%)	2 (20%)	0.007*
***bla***_**OXA−48**_	22 (52.3%)	7 (46.7%)	0.6	18 (58.1%)	11 (42.3%)	0.2	28 (59.6%)	1 (10%)	0.004*
***bla***_**KPC**_	10 (23.8%)	2 (13.3%)	0.6	5 (16.1%)	7 (26.9%)	0.5	10 (21.3%)	2 (20%)	0.9
***bla***_**IMP**_	5 (11.9%)	2 (13.3%)	0.7	5 (16.1%)	2 (7.7%)	0.3	7 (14.9%)	0 (0%)	0.2
***bla***_**GES**_	3 (7.1%)	0 (0%)	0.7	2 (6.4%)	1 (3.8%)	0.8	3 (6%)	0 (0%)	0.5
***bla***_**NDM**_	2 (4.8%)	0 (0%)	0.8	2 (6.4%)	0 (0%)	0.6	2 (4%)	0 (0%)	0.7
**Total** ^**+**^	38 (90.4%)	11 (73.3%)	0.2	28 (90.3)	21 (80.7)	0.5	44 (93.6)	5 (50)	0.005*
***IntI1***	26 (61.9%)	11 (73.3%)	0.4	23 (74.2)	14 (53.8)	0.1	35 (74.5)	2 (20)	0.001*
***IntI2***	7 (16.7%)	3 (20.0%)	0.7	4 (12.9)	6 (23.1)	0.3	9 (19.1)	1 (10)	0.4
**Tn** ***1999***	22 (52.3%)	7 (46.7%)	0.6	18 (58.1%)	11 (42.3%)	0.2	28 (59.6%)	1 (10%)	0.004*

P^1^, P-value between wound and urine isolates; P^2^, P-value between outpatients and inpatients isolates, P^3^, P-value between MDR and non-MDR isolates; Total^+^, total number of isolates contained one or more carbapenemase-encoding genes; * Significant level of P-value is < 0.05; P value was calculated by using Chi-square test

**Table 4:** Antibiotic resistance pattern of integron positive and integron negative carbapenem resistant *P*. *aeruginosa* isolates

Antibiotics	Total isolates (*N* = 57)	Integron-positive (*N* = 47)	Integron-negative (*N* = 10)	*P* value
	*N* (%)			
**Ceftazidime**	51 (89.5)	46 (97.9)	5 (50.0)	0.000*
**Cefepime**	33 (57.9)	30 (63.8)	3 (30.0)	0.049*
**Gentamycin**	25 (43.9)	19 (40.4)	6 (60.0)	0.2
**Amikacin**	28 (49.1)	27 (57.4)	1 (10.0)	0.006*
**Levofloxacin**	31 (54.4)	27 (57.4)	4 (40.0)	0.3
**Piperacillin –Tazobactam**	48 (84.2)	43 (91.5)	5 (50.0)	0.001*

* Significance level of P-value is < 0.05

* P-value was calculated by using Chi-square test

### Characterization of integrons and the associated gene cassettes among the tested isolates

**Fig. 1:**
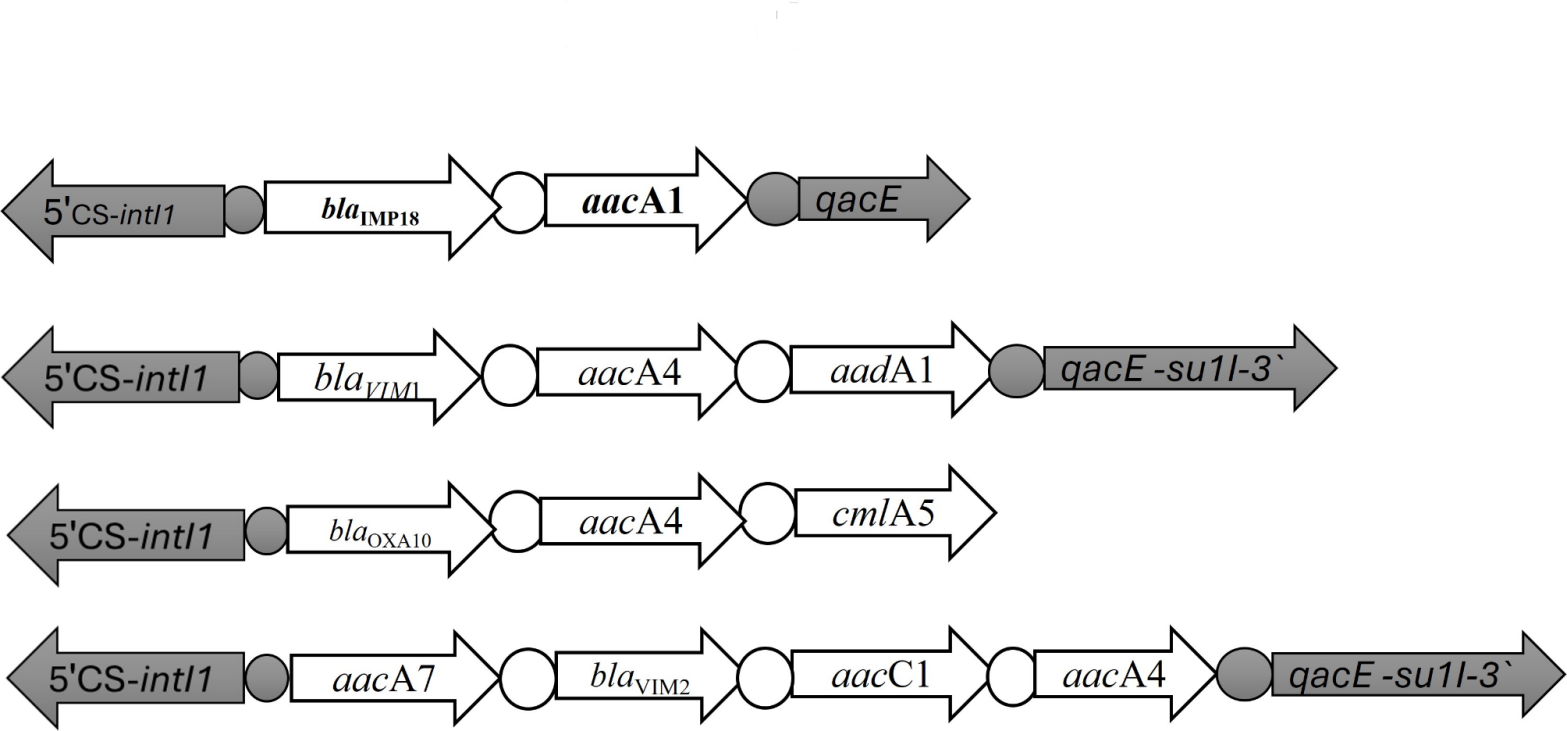
Schematic representation of gene cassette array located in the class 1 integrons isolated from 10 carbapenem-resistant *P. aeruginosa* clinical isolates. The horizontal arrows indicate the translation orientation of the genes. *attI* and *attC* (integron-associated recombination site) were shown as white and black circles, respectively

The characterization of the variable regions of the class 1 integrons amplified from the ten representative isolates previously identified as being positive for 3 carbapenemase-encoding genes was summarized in Table 5. All the tested isolates were MDR. The conserved segment of the tested integrons contained the integrase gene (*intI*) and the recombination site *attI1*. The variable segments of some tested class 1 integrons carried the antiseptic-resistance *qacE1* gene which confers resistance to quaternary ammonium compounds and the *sul1* gene which confers resistance to sulfonamides as shown in Fig. 1. All the tested integrons carried more than one resistance gene cassette as shown in Fig. 1. Three integrons contain two resistance gene cassettes, three integrons contain three gene cassettes, and 4 integrons contain up to four gene cassettes. The most prevalent gene cassette was: *aac*A7 | *bla*_VIM−*2*_ | *aac*C1 | *aac*A4 (4/10) followed by: *bla*_IMP−18_ | *aac*A1 (3/10), *bla*_VIM−1_ | *aac*A4 | *aad*A1 (2/10) and *bla*_OXA−10_ | *aac*A4 | *cml*A5 (1/10). All the tested integrons contained one carbapenemase-encoding gene cassette *(bla*_VIM_, *bla*_IMP,_*or bla*_*OXA−10*_).

**Table 5:** Characterization of the ten representative MDR isolates and their class 1 integrons

Isolate ID	Patient	Source	Carbapenemase genes detected	Gene cassette array	Antibiotic resistance profile
Pa 1	Inpatient	Wound	*bla*_VIM_, *bla*_OXA−48_, *bla*_KPC_	*aac*A7*-bla*_VIM2_ -*aac*C1*-aac*A4	CEZ, GM, AK, IMP, LEV
Pa13	Inpatient	Wound	*bla*_VIM_, *bla*_OXA−48_, *bla*_KPC_	*aac*A7*-bla*_VIM2_ -*aac*C1*-aac*A4	CEZ, CPM, AK, TZP, IMP
Pa19	Inpatient	urine	*bla*_VIM_, *bla*_OXA−48_, *bla*_KPC_	*bla*_VIM1_- *aac*A4*- aad*A1	CEZ, CPM, IMP, GM, TZP
Pa22	Outpatient	Wound	*bla*_KPC_, *bla*_OXA−48_, *bla*_NDM_,	*bla*_OXA10_-*aac*A4-*cml*A5	GM, CEZ, IMP, LEV, TZP
Pa25	Outpatient	Wound	*bla*_OXA−48_, *bla*_IMP_, *bla*_GES_	*bla*_IMP18_- *aac*A1	CEZ, CPM, GM, TZP, IMP
Pa33	Outpatient	Urine	*bla*_KPC_, *bla*_IMP_*bla*_OXA−48_	*bla*_IMP18_ | *aac*A1	CEZ, CPM, GM, AK, IMP
Pa43	Inpatient	Wound	*bla*_VIM_, *bla*_OXA−48_, *bla*_KPC_	*aac*A7-*bla*_VIM2_ -*aacC*1-*aacA*4	CEZ, CPM, GM, AK, IMP
Pa54	Inpatient	Urine	*bla*_OXA−48_, *bla*_IMP_, *bla*_NDM_	*bla*_IMP18_ | *aac*A1	CEZ, CPM, AK, IMP
Pa61	Outpatient	Wound	bla_VIM_, *bla*_KPC_, *bla*_OXA−48_	*aac*A7-*bla*_VIM2_ -*aac*C1-*aacA*4	GM, CEZ, IMP, LEV
Pa79	Outpatient	Wound	*bla*_VIM_, *bla*_KPC_, *bla*_OXA−48_	*bla*_VIM1_- *aac*A4*- aad*A1	CEZ, CPM, IMP, GM, TZP

Antibiotic abbreviations: Cefepime (CPM), Ceftazidime (CEZ), Gentamycin (GM), Amikacin (AK), Levofloxacin (LEV), Imipenem (IMP), and Piperacillin-Tazobactam (TZP). *bla*_OXA−48_ gene is associated with IS*1999* gene of Tn*1999*

### Detection of transposon Tn*1999* among carbapenem resistance *P. aeruginosa* clinical isolates

Conventional PCR was performed on all carbapenem-resistance *P. aeruginosa* clinical isolates using primers targeting Tn*1999* with an amplification product of 487 bp (supplementary figure S3). All the *bla*_OXA−48_-gene-positive *P. aeruginosa* clinical isolates were also positive for the amplified product and all the *bla*_OXA−48_*-*gene-negative *P. aeruginosa* clinical isolates were also negative for the amplified product.

## Discussion

There are restricted therapeutic choices available for treating infections caused by *P. aeruginosa.* Carbapenems, the last line of available antibiotics, is the drug of choice for treating resistant infections caused by these bacteria. Unfortunately, resistance to carbapenem has been emerging globally and becoming a public health threat. In this study, the resistance rate of our isolates to imipenem was high (58.8%). In line with our findings, several previous studies in Egypt revealed a higher resistance rate of carbapenem resistance among *P. aeruginosa* clinical isolates [19–21]. Certain factors are significantly associated with the emergence of carbapenem-resistant *P. aeruginosa* isolates including the development of MDR strains, prolonged hospitalization in burn units, and the abuse of carbapenem antibiotics in hospitals.

Carbapenem resistance among *P. aeruginosa* isolates is attributed mainly to carbapenemase production [22]. In this study, 86% of the carbapenem-resistant *P. aeruginosa* isolates carry carbapenemase genes, with *bla*_VIM_ being the most common gene. Numerous previous studies reported that *bla*_VIM_ is the most frequent carbapenemase-encoding gene detected in carbapenem-resistant *P. aeruginosa* clinical isolates [23–26]. In this study, out of 57 imipenem-resistant *P. aeruginosa* isolates, 29 (50.8%) isolates carried the *bla*_OXA−48_ gene. This frequency is slightly lower than that of Ali and Mohamed from Sudan who reported that out of 81 carbapenem-resistant isolates, 54 (66.7%) were positive for *bla*_OXA−48_ gene [27] and higher than that of Bahrami et al. in Iran who observed that the frequency of the *bla*_OXA−48_ gene was 12.5% among the studied carbapenem-resistant *P. aeruginosa* isolates [28].

OXA-type carbapenemases are rarely identified in *P. aeruginosa*, Carbapenem-resistant _OXA−48_ positive *P. aeruginosa* was recently reported in Sudan, India, Iran, and Turkey [28–30]. However, this is the first report identifying *bla*_OXA−48_-producing *P. aeruginosa* in Egypt. We suggest the carbapenem resistance genes were transmitted to *P. aeruginosa* via mobile genetic elements from other microorganisms such as Enterobacteriaceae and *A. baumannii* colonizing in burn units that implement poor infection control practical measures. Integrons are mobile genetic elements involved in the transfer of antimicrobial-resistant genes via horizontal gene transfer so, it’s important to understand the molecular epidemiology of clinically relevant and multi-drug-resistant bacteria. The integrons screening in this study reveals that the majority (64.9% and 74.5%, respectively) of carbapenem-resistant and MDR *P. aeruginosa* isolates carried class 1 integrons indicating the important role of these integrons in the acquisition and dissemination of carbapenem-resistance genes among pathogens. This finding agreed with a study by Yalda et al. in which the authors reported that the prevalence of class 1 integrons among carbapenem resistant *P. aeruginosa* isolates was 60% [31]. The overall prevalence of class 1 integron among *P. aeruginosa* isolates in this study was 54.6%, this is in concordance with numerous published studies on different clinical samples reported that the prevalence of integrons among *P. aeruginosa* isolates was high and most of them reported a higher incidence of class 1 integrons [32–34]. However, the prevalence rate of class 1 integrons detected in this study is higher than that of Fonseca et al., 2005 in which the rate was 41.5% [35], and Xu et al. reported a rate of 45.8% [36]. The incidence of class 1 integrons detected in this study and the other recent studies was higher than the older ones, this was comparable with the continuously increasing resistance to carbapenem. This difference indicates a high risk of resistant genes-producing isolates in hospitals following the dissemination of integrons. Hence, antibiotic prescribing guidelines and infection control measures must be firmly enforced to overcome the increasing threat. The overall prevalence of class 2 integrons among the studied *P. aeruginosa* isolates was 12.4%. In line with our findings, several previous studies reported a low rate of class 2 integrons in *P. aeruginosa* clinical isolates [17, 31, 37]. The lower incidence rate of class 2 integrons indicates the restricted distribution of these genetic elements among bacterial populations. In this study, the resistance rates of integron-positive isolates to ceftazidime, cefepime, amikacin, and Piperacillin –Tazobactam were significantly higher than integron-negative isolates (*P <* 0.05). This agreed with a study done by Wang et al. who reported that the antibiotic-resistant rates among the isolates containing integrons were significantly higher than those without integrons (*P <* 0.05) [38]. To further identify the role of integron class 1 in antibiotic resistance transmission and dissemination and following the higher prevalence of *bla*_OXA−48_ gene and integron class 1 among carbapenem-resistant isolates in this study, the PCR products of the integron genes from 10 MDR isolates containing up to 3 carbapenemase encoding genes were sequenced to detect the associated antibiotic-resistant gene cassettes. In the present study, 4 types of resistant gene cassettes were observed in 10 different integrons variable regions of the PCR products. The detected gene cassettes encode mainly resistance to aminoglycosides antibiotics including amikacin (*aac*A1, *aac*A4, *aac*A7, *aac*C1, and *aad*A1) and beta-lactam antibiotics including carbapenems (*bla*_VIM_, *bla*_IMP_ or *bla*_OXA10_), this explains the wide range of antibiotic resistance among these isolates. All the tested integrons contained one carbapenem-resistant gene cassette. In this study, we couldn’t identify any resistant gene cassette on the tested integrons for the *bla*_OXA−48_ gene. While all the *bla*_OXA−48_ gene-positive isolates contained the Tn*1999* transposon gene and the Tn*1999* gene was absent in all *bla*_OXA−48_ negative isolates. Concordance with previously published studies that reported the *bla*_OXA−48_ gene had been identified in association with Tn*1999*, providing promoter sequences, IS*1999* responsible for its expression [4, 39, 40]. One of the limitations of this study is that all the isolates were from the same region, further multi-centric studies with large sample sizes are recommended to understand the characterization and the epidemiology of these isolates. Furthermore, we couldn’t perform whole genome sequencing of all isolates due to a lack of funds in this study.

## Conclusions

There is a high risk of antibiotic resistance transmission and dissemination of integrons-positive isolates in hospitals. Our data demonstrated a high incidence of class 1 integrons and carbapenemase encoding genes, *bla*_VIM,_ and *bla*_OXA−48_ genes in carbapenem-resistant *P. aeruginosa* strains isolated from wound and urine samples. Moreover, all the *bla*_OXA−48_-gene-positive *P. aeruginosa* clinical isolates were positive for the Tn*1999* transposon gene. Therefore, mobile genetic elements have an important role in carbapenem-resistance gene transfer among pathogens. The management of infection control policies and the appropriate use of antibiotics are essential for controlling the spreading of antibiotic resistance.

## Electronic Supplementary Material

Below is the link to the electronic supplementary material.

Supplementary Material 1

## Data Availability

Data is provided within the manuscript or supplementary information file.
