# Supplementary material for: Prevalence of blaOXA-48 and other carbapenemase encoding genes among carbapenem-resistant Pseudomonas aeruginosa clinical isolates in Egypt
Source: BMC Infect Dis. 2024 Nov 11;24:1278. doi: 10.1186/s12879-024-10123-7 (PMC11556172; doi:10.1186/s12879-024-10123-7)
Supplement: Supplementary file 1 — Supplementary Material 1 [file 12879_2024_10123_MOESM1_ESM.docx]

**Supplementary Table S1: Sequences of primers used in this study.**

| Gene | Primer sequence | Annealing temp (°C) | Product size (bp) |
| --- | --- | --- | --- |
| *16S rRNA* | F-TCAACCTGGGAACTGCATCC  R-AGGCACCAATCCATCTCTGG | 55 | 410 |
| Class A β-lactamases | | | |
| *bla*KPC | F-TGTTGCTGAAGGAGTTGGGC  R-ACGACGGCATAGTCATTTGC | 56 | 340 |
| *bla*GES | F-GAAACCAAACGGGAGACGC  R-CTTGACCGACAGAGGCAACT | 60 | 207 |
| *bla*SME | F-TATGGAACGATTTCTTGGCG  R-CTCCCAGTTTTGTCACCTAC | 56 | 300 |
| Class B β-lactamases | | | |
| *bla*IMP | F-GGAATAGAGTGGCTTAAYTCTC  R- GGTTTAAYAAAACAACCACC | 50 | 232 |
| *bla*VIM | F- CCGTGATGGTGATGAGTTGC  R- CTACTGGACCGAAGCGCAC | 55 | 290 |
| *bla*NDM | F- TTGCGACTTATGCCAATGCG  R- CAGCCACCAAAAGCGATGTC | 56 | 214 |
| Class D β-lactamases | | | |
| *bla*OXA-48 | F-GCTTGATCGCCCTCGATT  R-GATTTGCTCCGTGGCCGAAA | 60 | 281 |
| Integrons | | | |
| *intI1 Class 1 Integrase* | F-GGTCAAGGATCTGGATTTCG  R-ACATGCGTGTAAATCATCGTC | 50 | 483 |
| *intI2 Class 2 Integrase* | F-CACGGATATGCGACAAAAAGG T  R-GTAGCAAACGAGTGACGAAATG | 59 | 789 |
| *intI3 Class 3 Integrase* | F-AGTGGGTGGCGAATGAGTG  R-TGTTCTTGTATCGGCAGGTG | 62 | 600 |
| *Class 1 Integron variable region* | 5′-CS: GGCATCCAAGCAGCAAG  3′-CS: AAGCAGACTTGACCT`GA | 55 | Variable |
| IS1999 | F-ATCGGATGGTTTAGGGCTGC  R-CTACGGGGTGTATTCGAGCC | 56 | 487 |

*
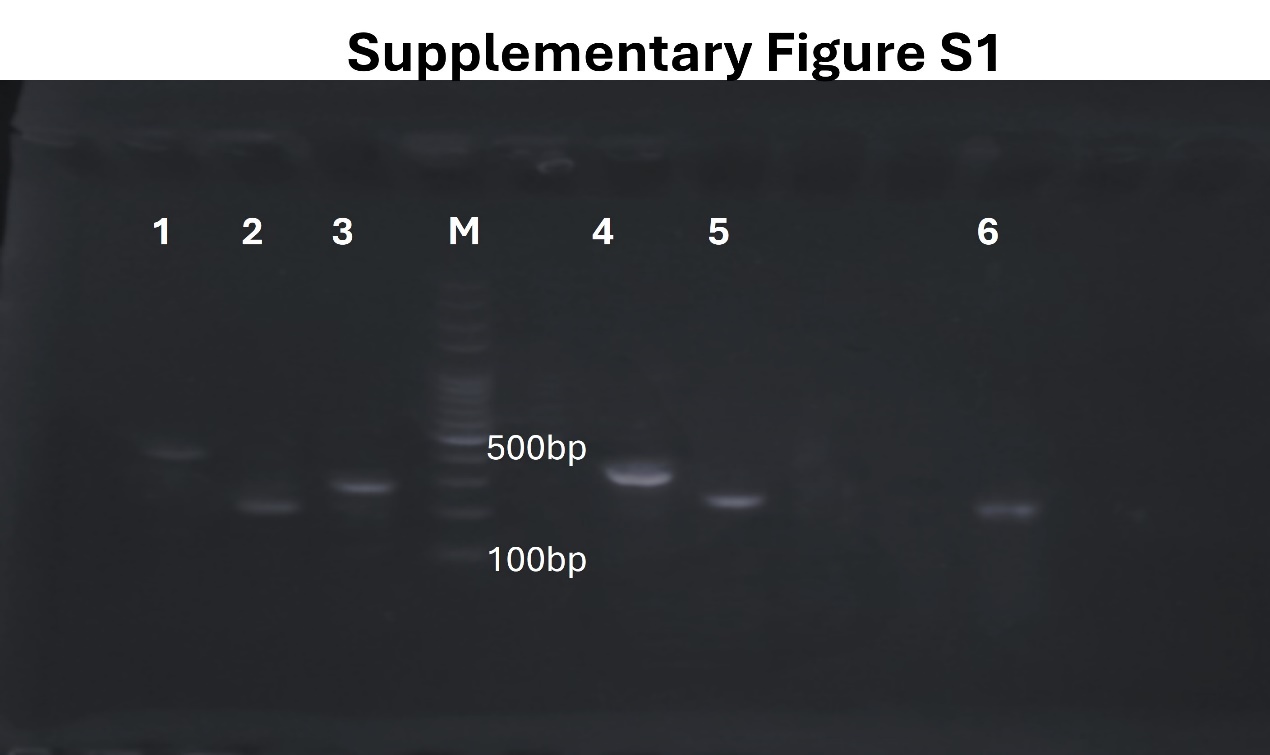
*

**Supplementary Figure S1: Gel electrophoresis of the PCR amplified products for the tested genes**. Lane M is a molecular size marker L(100-1000bp). Lane 1: *16S rRNA*(410 bp), Lane 2: *bla*GES (207 bp); Lane 3: *bla*VIM (290 bp); Lane 4: *bla*KPC (340 bp); Lane 5: *bla*IMP (232 bp); Lane 6: *bla*NDM (214 bp).


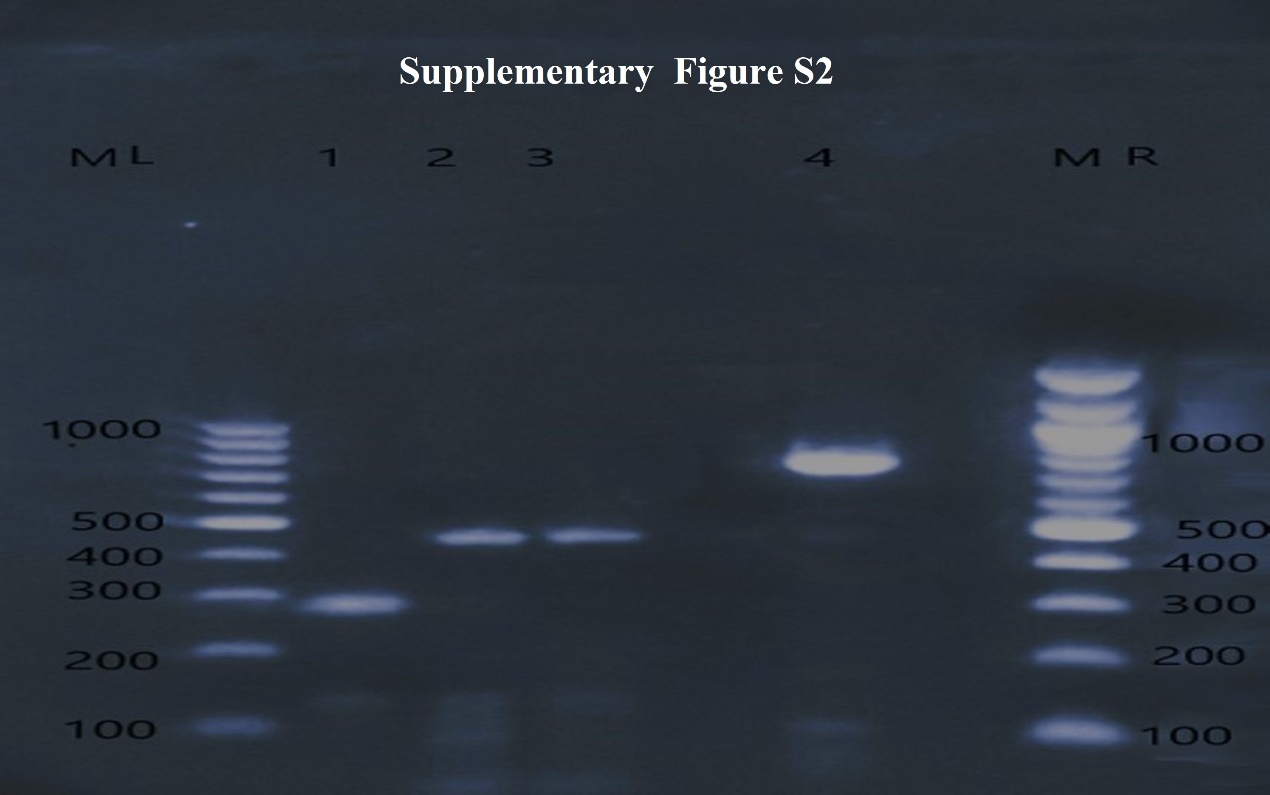


**Supplementary Figure S2: Gel electrophoresis of the PCR amplified products for the tested genes**. Lane M is a molecular size marker L(100-1000bp) and R(100-2000). Lane 1: PCR product of *bla* OXA-48 gene (281 bp), Lane 2 and 3, PCR product of *intI1*gene (483 bp); Lane 4, PCR product of *intI2* gene (789 bp).


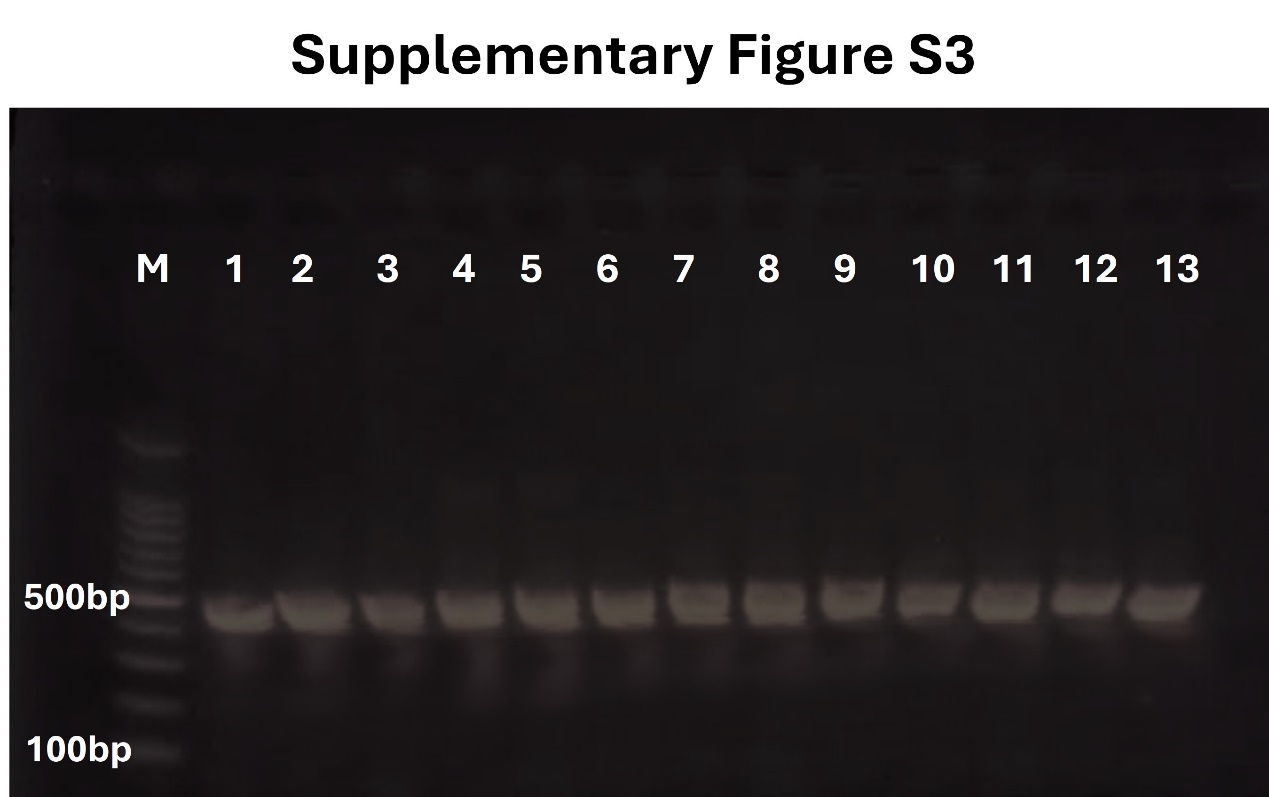


**Supplementary Figure S3: Gel electrophoresis of the PCR amplified products for the tested genes**. Lane M is a molecular size marker L(100-1000bp); Lane 1-13: PCR product of Tn1999 gene (487 bp).
